# Synthesis and Biological Evaluation of Novel 4-Morpholino-7,8-dihydro-5*H*-thiopyrano[4,3-*d*]pyrimidine Derivatives Bearing Phenylpyridine/ Phenylpyrimidine-Carboxamides

**DOI:** 10.3390/molecules21111447

**Published:** 2016-10-31

**Authors:** Huimin Liu, Wenhui Wang, Chengyu Sun, Caolin Wang, Wufu Zhu, Pengwu Zheng

**Affiliations:** 1 School of Perfume and Aroma Technology, Shanghai Institute of Technology, Shanghai 201418, China; szliuhm@sit.edu.cn; 2 School of Pharmacy, Jiangxi Science & Technology Normal University, Nanchang 330013, China; wenhui410@126.com (W.W.); sunchengyu0902@126.com (C.S.); wangcllw@126.com (C.W.); 3 Pharmacy Department, The Affiliated Hospital of Chongqing Three Gorges Medical College, Chongqing 404000, China

**Keywords:** thiopyrano[4,3-*d*]pyrimidine, phenylpyridine/phenylpyrimidine carboxamides, synthesis, cytotoxicity activity, PI3Kα kinase

## Abstract

Four series of novel 4-morpholino-7,8-dihydro-5*H*-thiopyrano[4,3-*d*]pyrimidine derivatives **11a**–**j**, **12a**–**j**, **13a**–**g** and **14a**–**g** bearing phenylpyridine/phenylpyrimidine- carboxamide scaffolds were designed, synthesized and their IC_50_ values against three cancer cell lines (A549, PC-3 and MCF-7) were evaluated. Eleven of the compounds showed moderate cytotoxicity activity against the cancer cell lines. Structure-activity relationships (SARs) and pharmacological results indicated that the introduction of phenylpyridine-carboxamide scaffold was beneficial for the activity. What’s more, the oxidation of the sulfur atom in thiopyran and various types of substituents on the aryl group have different impacts on different series of compounds. Furthermore, the positions of aryl group substituents have a slight impact on the activity of the phenylpyridine-carboxamide series compounds.

## 1. Introduction

**Figure 1:**
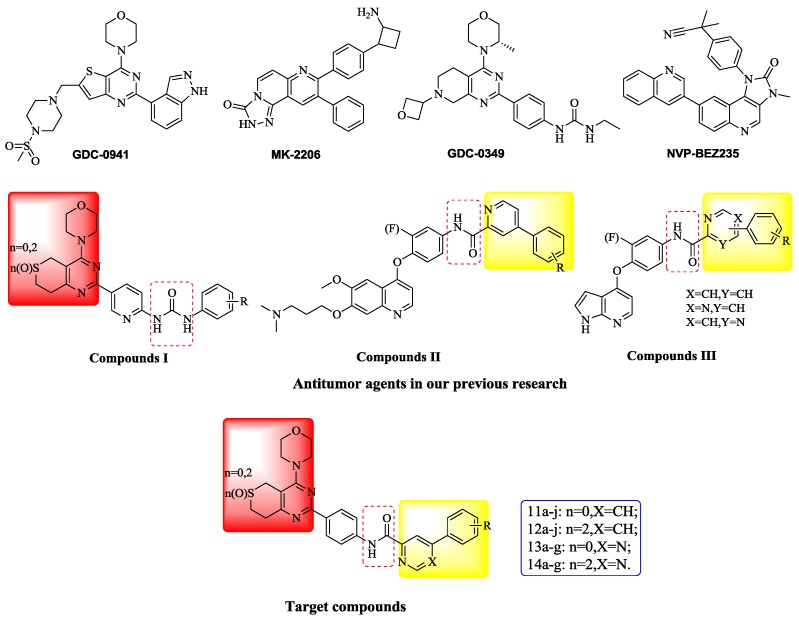
Structures of representative compounds of four kinds of inhibitors and target compounds.

The PI3K/AKT/mTOR signaling cascade is one of the most important intracellular pathways, which is frequently activated in different types of cancer [1]. Inhibition of the PI3K/Akt/mTOR pathway can effectively block the abnormal signal transduction of various growth factors leading to suppression of the occurrence and development of cancer, so it has become a hotspot of cancer prevention and cancer treatment research. Currently, PI3K/Akt/mTOR pathway inhibitors include PI3K inhibitors, Akt inhibitors, mTOR inhibitors and dual PI3K/mTOR inhibitors and representative compounds are GDC-0941, MK-2206, GDC-0349 and NVP-BEZ235, respectively [2] (Figure 1).

According to our previous study [3,4,5], we modified some 4-morpholino-7,8-dihydro-5*H*-thiopyrano [4,3-*d*]pyrimidine derivatives. Firstly, the urea group of compounds I were replaced with an amide scaffold according to the theory of bioisosterism. Secondly, different aromatic heterocycles were introduced on the carboxy-terminal of the amide inspired by compounds II and III. In addition, various R groups were introduced on the benzene ring to investigate their impact on activity. As a result, four series of 4-morpholino-7,8-dihydro-5*H*-thiopyrano[4,3-*d*]pyrimidine derivatives bearing phenylpyridine/phenylpyrimidine carboxamide scaffolds were designed as PI3K inhibitors and synthesized.

Herein we disclose the synthesis and antitumor activity of all the target compounds against the A549 (human lung cancer), PC-3 (human prostatic cancer) and MCF-7 (human breast cancer) cancer cell lines, and the anti-PI3Kα kinase activity of one selected compound.

## 2. Results and Discussion

**Scheme 1:**
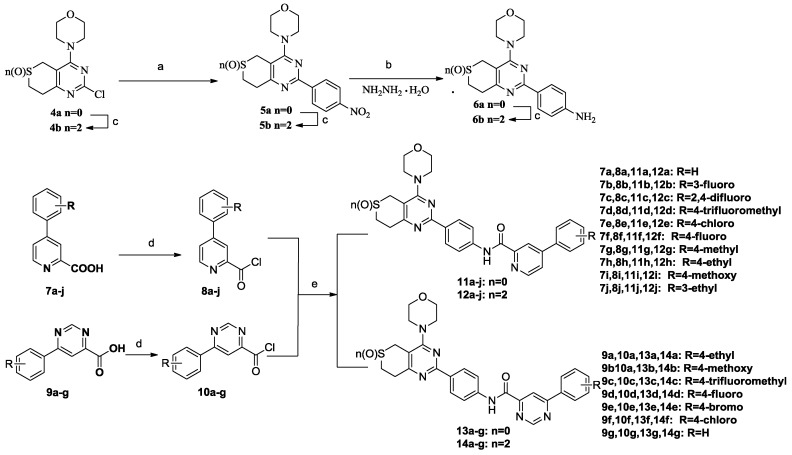
Synthetic routes to the target compounds. *Reagents and conditions*: (a) (1) 1-bromo-4-nitro-benzene, bis(pinacolato)diboron, KAc, Pd(PPh_3_)Cl_2_, 1,2-dimethoxyethane, reflux, 2 h; (2) H_2_O, Na_2_CO_3_, Pd(PPh_3_)_2_Cl_2_, reflux, 6 h; (b) 80% NH_2_NH_2_·H_2_O, FeCl_3_·6H_2_O, actived C, EtOH, 78 °C, 1 h; (c) Na_2_WO_4_·2H_2_O, 30% H_2_O_2_, 20 °C, 3 h; (d) (COCl)_2_, DMF, CH_2_Cl_2_, r.t., 0.5 h; (e) DIPEA, CH_2_Cl_2_, r.t., 0.5 h.

The preparation of target compounds **11a**–**j**, **12a**–**j**, **13a**–**g** and **14a**–**g** is described in Scheme 1. Compounds **4a** and **4b** were synthesized according to the reported procedures [3,6,7]. Compounds **6a** and **6b** were synthesized from **4a** or **4b** and 1-bromo-4-nitrobenzene through Suzuki-coupling reactions, followed by reduction with 80% NH_2_NH_2_·H_2_O. Compounds **7a–j** and **9a–g** were synthesized according to the reported procedures [4,5,8]. Carbonyl chlorides **8a–j** and **10a–g** were obtained by **7a–j** and **9a–g** throng Chlorination. Then, amides **6a** and **6b** were reacted with phenylpyridine carbonyl chlorides **8a**–**j** or phenylpyrimidine carbonyl chlorides **10a**–**g** which were synthesized according to the reported procedures [6,7,9] through promotion by DMF and DIPEA in dichloromethane to yield the target compounds **11a**–**j**, **12a**–**j**, **13a**–**g** and **14a**–**g**, respectively.

### 2.1. Biological Evaluation

Taking GDC-0941 as reference compound, the target compounds **1a**–**j**, **12a**–**j**, **13a**–**g** and **14a**–**g** ere evaluated for the cytotoxicity against the three cancer cell lines A549, PC-3 and MCF-7 usg 3-(4,5-dimethylthiazolyl-2)-2,5-diphenyltetrazolium bromide (MTT) cell proliferation assays. In addition, one selected compounds **12h** was fher evaluated for PI3Kα kinase inhibitory activity. The results, expressed as IC_50_ values or inhibitory rate, are sumrized in Table 1, Table 2 and Table 3.

As shown in Table 1 and Table 2, eleven of the compounds showed moderate cytotoxicity activity against the three cancer cells. In the first series (compounds **11a**–**j**), compounds substituted at the aryl group C-3 position displayed better activity than those substituted at the C-4 position, as seen by comparing **11b** (IC_50_ values: 48.08 ± 0.13 µM, 60.29 ± 1.24 µM, 51.67 ± 1.56 µM) and **11f** (no activity against all three cancer lines) or **11j** (IC_50_ values: 11.59 ± 0.11 µM, 15.29 ± 0.83 µM, 12.43 ± 0.96 µM) and **11h** (no activity against all three cancer lines). What’s more, the introduction of electron-donating groups at the C-3 position was likely to enhance the activity, as inferred from **11b** and **11j**. The substituents at the C-4 position had no significant impact on the activity. In the second series (compounds **12a**–**j**), the presence of electron withdrawing groups (-Cl, -F, -CF_3_) cancelled the activity of the target compounds against all three cancer lines, as exemplified by **12b**–**f**. In this case we inferred that compounds substituted at the C-4 position of aryl group might revealed better activity than those substituted at C-3 position by comparing **12h** (IC_50_ values: 8.37 ± 0.10 µM, 11.34 ± 0.11 µM, 9.26 ± 0.81 µM) and **12j** (no activity against all three cancer lines). In addition, the presence of electron-donating groups such as methyl and ethyl at the aryl group C-4 position might be appropriate to increase the activity. In the third series (compounds **13a**–**g**), the activity against all the three cancer lines of all the target compounds had vanished. In the fourth series (compounds **14a**–**g**), the compounds with individual substituents such as ethyl, methoxy and bromo showed moderate cytotoxicity activity against the MCF-7 cell line.

By comparison, the target compounds bearing phenylpyridine carboxamide scaffold (i.e., **11a–j** and **12a–j**) showed better activity than those compounds bearing a phenylpyrimidine carboxamide scaffold (compounds **13a**–**g** and **14a**–**g**). What’s more, the oxidation of the sulfur atom played no significant impact on the activity in the first two series. However, it seemed to increase the activity in the last two series of compounds. Finally the inhibitory rate against PI3Ka kinase of the selected compound **12h** at 10 µM was further examined. It exhibited a moderate inhibitory rate against PI3Ka kinase.

### 2.2. Molecular Docking Study

To explore the binding modes of target compounds with the active site of PI3Ka, molecular docking simulation studies were carried out by using SURFLEX-DOCK module of SYBYL package version. Based on the in vitro inhibition results, we selected compound **12h** as ligand examples, and the structures of PI3Ka (PDB ID code: 4L23 [9]) were selected as the docking models.

**Figure 2:**
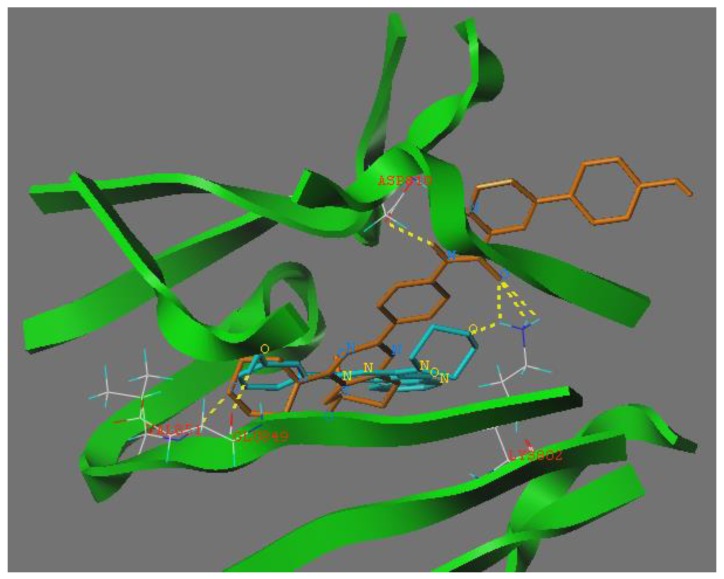
Binding models of compound **12h** (shown in orange sticks) and native ligand PI 103 (shown in blue sticks) with PI3Ka. The proteins were displayed by green ribbon. Hydrogen bonds were showed in dashed lines (yellow).

The binding modes of compound **12h** with the active site of PI3Ka molecular are shown in Figure 2, where as depicted the morpholino groups of compounds **12h** and PI103 were almost completely overlapped. In the docking model of compound **12h** with PI3Ka, we can see the morpholino group formed one hydrogen bond with the VAL815 residue. In addition, the nitrogen atom of the amide group formed one hydrogen bond with the ASP810 residue and the oxygen atom of the amide group also formed three hydrogen bonds with residue LYS802, respectively. The abovementioned SAR analysis and molecular docking study results may allow the rational design of potential PI3K inhibitors.

## 3. Experimental Section

### 3.1. General Information

All melting points were obtained on a Büchi Melting Point B-540 apparatus (Büchi Labortechnik, Flawil, Switzerland) and were uncorrected. NMR spectra were recorded using a Bruker 400 MHz spectrometer (Bruker Bioscience, Billerica, MA, USA) with TMS as an internal standard. Mass spectra (MS) were taken in ESI mode on an Agilent 1100 LCMS (Agilent, Palo Alto, CA, USA). TLC analysis was carried out on silica gel plates GF254 (Qingdao Haiyang Chemical, Qingdao, China). All the materials were obtained from commercial suppliers and used without purification, unless otherwise specied. Yields were not optimized.

### 3.2. Chemistry

#### 3.2.1. General Procedure for the Preparation of Compounds **4a**,**b**, **5a**,**b** and **6a**,**b**

Compounds **4a,b** were synthesized according to the procedures previously reported by our research group [3,6,7]. A solution of 1-bromo-4-nitrobenzene (5.0 g, 0.029 mol), bis(pinacolato) diboron (11.1 g, 0.043 mol), potassium acetate (8.7 g, 0.089 mol) and bistriphenylphosphine palladium dichloride (0.5 g, 0.7 mmol) was added to 1,4-dioxane (250 mL) and heated to 110 °C. The reaction was continued for 2.5 h under N_2_ and monitored by TLC. H_2_O (40 mL) was added to the solution and continued for 5 min. Subsequently, **4a** or **4b** (5.9 g, 0.021 mol), Na_2_CO_3_ (4.7 g, 0.044 mol) and bistriphenylphosphine dichloride (0.5 g, 0.7 mmol) were added successively. The reaction was continued for about 6 h. The mixture was cooled and concentrated under vacuum. H_2_O (250 mL) was added, stirred for 30 min and then filtered. The filter cake was decolorized with activated carbon (5 g) and silica gel (2 g) in a mixed solvent (CH_2_Cl_2_:CH_3_OH = 4:1, 500 mL), filtered while hot and concentrated under vacuum to obtain **5a** or **5b** which was recrystallized from ethanol.

Then **5a** or **5b** (0.020 mol) was refluxed with hydrazine hydrate (0.2 mol), Ferric chloride (0.01 mol) and an appropriate amount of activated carbon in ethanol (100 mL) for 10 min and monitored by TLC. The reaction solution was cooled. Then the insoluble solid was filtered, the filtrate was evaporated, after that water was added and stirred at room temperature for 30 min. Afterwards, the yellow solid was filtered off and dried to obtain the desired target compounds **6a** or **6b**.

#### 3.2.2. General Procedure for the Preparation of Compounds **7a**–**j**, **8a**–**j**, **9a**–**g** and **10a**–**g**

Compounds **7a**–**j** and **9a**–**g** were synthesized according to the procedures reported by our research group [4,5,8]. Then compounds **7a**–**j** or **9a**–**g** (0.02 mmol) were dissolved in thionyl chloride (8 mL) and refluxed for 1 h. The reaction mixture was evaporated to yield the corresponding chloride which was dissolved in dichloromethane (10 mL). The solution (**8a**–**j** or **10a**–**g**) was used for the next step without further purification.

#### 3.2.3. General Procedure for the Preparation of Target Compounds **11a**–**j**, **12a**–**j**, **13a**–**g** and **14a**–**g**

A solution of phenylpyrimidine chloride (0.01 mol, **8a**–**j** and **10a**–**g**) in dichloromethane (10 mL) was added dropwise to a solution of **6a** or **6b** (0.075 mol) and *N*,*N*-diisopropylethylamine (0.03 mol) in dichloromethane (10 mL) in an ice bath. Upon completion of the addition, the reaction mixture was removed from the ice bath, held at room temperature for 15 min and monitored by thin-layer chromatography (TLC). The mixture was washed with 10% K_2_CO_3_ (50 mL × 3) followed by brine (50 mL × 1), and the organic phase was separated, dried over anhydrous sodium sulfate, and evaporated to yield the target compounds **11a**–**11j**, **12a**–**12j**, **13a**–**13g** and **14a**–**14g**, which were recrystallized from isopropanol.

*N-(4-(4-Morpholino-7,8-dihydro-5H-thiopyrano[4,3-d]pyrimidin-2-yl)phenyl)-4-phenylpyridine carboxamide* (**11a**). A pale yellow solid; Yield: 66.8%; m.p.: 244.1–242.8 °C; ESI-MS [M + H] *m*/*z*: 510.2; ^1^H-NMR (CDCl_3_) δ 10.55 (s, 1H), 8.75 (d, *J* = 5.1 Hz, 1H), 8.71 (d, *J* = 8.3 Hz, 2H), 8.65 (s, 1H), 8.04 (d, *J* = 8.2 Hz, 2H), 7.77 (d, *J* = 3.8 Hz, 1H), 7.60 (d, *J* = 7.7 Hz, 1H), 7.52 (s, 1H), 7.52–7.48 (m, 1H), 7.47 (s, 1H), 7.20 (d, *J* = 2.1 Hz, 1H), 3.95 (s, 4H), 3.89 (s, 6H), 3.64 (s, 2H), 3.04 (s, 2H).

*N-(4-(4-Morpholino-7,8-dihydro-5H-thiopyrano[4,3-d]pyrimidin-2-yl)phenyl)-4-(3-fluorophenyl)pyridine amide* (**11b**). A pale yellow solid; Yield: 65.3%; m.p.: 236.5–238.4 °C; ESI-MS [M + H] *m*/*z*: 528.1; ^1^H-NMR (CDCl_3_) δ 11.02 (s, 1H), 8.95 (s, 1H), 8.82 (d, *J* = 5.4 Hz, 1H), 8.63 (d, *J* = 8.7 Hz, 2H), 8.08 (d, *J* = 8.7 Hz, 2H), 7.91 (d, *J* = 2.1 Hz, 2H), 7.90 (s, 1H), 7.56 (s, 1H), 7.54 (d, *J* = 1.8 Hz, 1H), 3.95 (s, 4H), 3.87 (s, 4H), 3.84 (s, 2H), 3.65 (s, 2H), 3.05 (d, *J* = 6.0 Hz, 2H).

*N-(4-(4-Morpholino-7,8-dihydro-5H-thiopyrano[4,3-d]pyrimidin-2-yl)phenyl)-4-(2,4-difluorophenyl) picolinamide* (**11c**). A pale yellow solid; Yield: 66.6%; m.p.: 235.0–236.7 °C; ESI-MS [M + H] *m*/*z*: 546.1; ^1^H-NMR (CDCl_3_) δ 10.42 (s, 1H), 8.73 (d, *J* = 3.1 Hz, 2H), 8.72 (s, 1H), 8.49 (s, 1H), 8.03 (d, *J* = 8.5 Hz, 2H), 7.74–7.71 (m, 1H), 7.61 (td, *J* = 8.7, 6.3 Hz, 1H), 7.07 (td, *J* = 8.1, 2.3 Hz, 1H), 7.00 (ddd, *J* = 11.0, 8.7, 2.5 Hz, 1H), 3.94 (s, 4H), 3.89 (s, 6H), 3.62 (s, 2H), 3.04 (s, 2H).

*N-(4-(4-Morpholino-7,8-dihydro-5H-thiopyrano[4,3-d]pyrimidin-2-yl)phenyl)-4-(4-trifluoromethylphenyl) picolinamide* (**11d**). A yellow solid; Yield: 64.9%; m.p.: 229.3–231.7 °C; ESI-MS [M + H] *m*/*z*: 578.2; ^1^H-NMR (DMSO-*d*_6_) δ 10.86 (s, 1H), 8.87 (s, 1H), 8.47 (s, 1H), 8.34 (d, *J* = 8.3 Hz, 2H), 8.13 (d, *J* = 7.5 Hz, 2H), 8.07 (s, 2H), 8.05 (s, 1H), 7.93 (d, *J* = 7.9 Hz, 2H), 3.76 (s, 4H), 3.71 (s, 2H), 3.39 (s, 4H), 3.07 (s, 2H), 3.00 (s, 2H).

*N-(4-(4-Morpholino-7,8-dihydro-5H-thiopyrano[4,3-d]pyrimidin-2-yl)phenyl)-4-(4-chlorophenyl)pyridine amide* (**11e**). A pale yellow solid; Yield: 65.1%; m.p.: 235.3–236.2 °C; ESI-MS [M + H] *m*/*z*: 545.2; ^1^H-NMR (CDCl_3_) δ 12.09 (s, 1H), 9.81 (s, 1H), 9.14 (s, 1H), 8.30 (s, 3H), 8.23 (s, 2H), 8.10 (s, 2H), 7.56 (s, 2H), 3.95 (s, 4H), 3.88 (s, 4H), 3.75 (s, 2H), 3.68 (s, 2H), 3.08 (s, 2H).

*N-(4-(4-Morpholino-7,8-dihydro-5H-thiopyrano[4,3-d]pyrimidin-2-yl)phenyl)-4-(4-fluorophenyl)pyridine amide* (**11f**). A yellow solid; Yield: 63.7%; m.p.: 248.1–249.6 °C; ESI-MS [M + H] *m*/*z*: 528.2; ^1^H-NMR (DMSO-*d*_6_) δ 10.84 (s, 1H), 8.82 (s, 1H), 8.43 (s, 1H), 8.35 (d, *J* = 8.3 Hz, 2H), 8.07 (d, *J* = 8.4 Hz, 2H), 8.01 (s, 3H), 7.43 (d, *J* = 8.8 Hz, 2H), 3.78 (s, 4H), 3.73 (s, 2H), 3.40 (s, 4H), 3.09 (s, 2H), 3.02 (s, 2H).

*N-(4-(4-Morpholino-7,8-dihydro-5H-thiopyrano[4,3-d]pyrimidin-2-yl)phenyl)-4-(4-methylphenyl) picolinamide* (**11g**). A pale yellow solid; Yield: 64.1%; m.p.: 275.0–276.8 °C; ESI-MS [M + H] *m*/*z*: 524.2; ^1^H-NMR (DMSO-*d*_6_) δ 10.84 (s, 1H), 8.79 (s, 1H), 8.42 (s, 1H), 8.35 (d, *J* = 8.0 Hz, 2H), 8.07 (d, *J* = 8.7 Hz, 2H), 8.02–7.98 (m, 1H), 7.83 (d, *J* = 7.3 Hz, 2H), 7.40 (d, *J* = 7.9 Hz, 2H), 3.78 (s, 4H), 3.73 (s, 2H), 3.41 (s, 4H), 3.09 (s, 2H), 3.02 (s, 2H), 2.41 (s, 3H).

*N-(4-(4-Morpholino-7,8-dihydro-5H-thiopyrano[4,3-d]pyrimidin-2-yl)phenyl)-4-(4-ethylphenyl) picolinamide* (**11h**). A pale yellow solid; Yield: 67.3%; m.p.: 229.8–232.1 °C; ESI-MS [M + H] *m*/*z*: 538.2; ^1^H-NMR (DMSO-*d*_6_) δ 10.82 (s, 1H), 8.79 (s, 1H), 8.41 (s, 1H), 8.33 (d, *J* = 8.4 Hz, 2H), 8.05 (d, *J* = 9.1 Hz, 2H), 7.99 (s, 1H), 7.83 (d, *J* = 8.0 Hz, 2H), 7.41 (d, *J* = 7.8 Hz, 2H), 3.76 (s, 4H), 3.71 (s, 2H), 3.37 (s, 4H), 3.08 (s, 2H), 3.00 (s, 2H), 2.69 (d, *J* = 7.5 Hz, 2H), 1.22 (s, 3H).

*N-(4-(4-Morpholino-7,8-dihydro-5H-thiopyrano[4,3-d]pyrimidin-2-yl)phenyl)-4-(4methoxyphenyl) pyridine-carboxamide* (**11i**). A pale yellow solid; Yield: 63.7%; m.p.: 233.6–235.1 °C; ESI-MS [M + H] *m*/*z*: 540.2; ^1^H-NMR (DMSO-*d*_6_) δ 10.84 (s, 1H), 8.76 (d, *J* = 5.2 Hz, 1H), 8.40 (d, *J* = 1.3 Hz, 1H), 8.36 (s, 1H), 8.34 (s, 1H), 8.08 (s, 1H), 8.06 (s, 1H), 7.98 (dd, *J* = 5.2, 1.9 Hz, 1H), 7.92–7.89 (m, 1H), 7.89 (d, *J* = 2.1 Hz, 1H), 7.14 (d, *J* = 5.4 Hz, 1H), 7.13 (s, 1H), 3.85 (s, 3H), 3.80–3.74 (m, 4H), 3.73 (s, 2H), 3.40 (s, 4H), 3.09 (t, *J* = 6.4 Hz, 2H), 3.01 (t, *J* = 6.3 Hz, 2H).

*N-(4-(4-Morpholino-7,8-dihydro-5H-thiopyrano[4,3-d]pyrimidin-2-yl)phenyl)-4-(3-methylphenyl)* *pyridine carboxamide* (**11j**). A white solid; Yield: 65.6%; m.p.: >300.0 °C; ESI-MS [M + H] *m*/*z*: 542.1; ^1^H-NMR (DMSO-*d*_6_) δ 10.83 (s, 1H), 8.80 (s, 1H), 8.41 (s, 1H), 8.34 (d, *J* = 8.1 Hz, 2H), 8.05 (d, *J* = 8.7 Hz, 2H), 8.00 (s, 1H), 7.72 (s, 1H), 7.67 (s, 1H), 7.46 (s, 1H), 7.35 (s, 1H), 3.76 (s, 4H), 3.71 (s, 2H), 3.39 (s, 4H), 3.07 (s, 2H), 3.00 (s, 2H), 2.43 (s, 3H).

*N-(4-(4-Morpholino-7,8-dihydro-6,6-dioxide-5H-thiopyrano[4,3-d]pyrimidin-2-yl)phenyl)-4-phenylpyridine Amide* (**12a**). A white solid; Yield: 61.9%; m.p.: 229.0–233.8 °C; ESI-MS [M + H] *m*/*z*: 524.1; ^1^H-NMR (DMSO-*d*_6_) δ 10.87 (s, 1H), 8.82 (s, 1H), 8.42 (s, 1H), 8.35 (d, *J* = 8.6 Hz, 2H), 8.08 (d, *J* = 8.4 Hz, 2H), 8.02 (s, 1H), 7.90 (d, *J* = 7.8 Hz, 2H), 7.57 (d, *J* = 7.4 Hz, 3H), 4.35 (s, 2H), 3.76 (s, 4H), 3.56 (s, 2H), 3.39 (s, 6H).

*N-(4-(4-Morpholino-7,8-dihydro-6,6-dioxide-5H-thiopyrano[4,3-d]pyrimidin-2-yl)phenyl)-4-(3-fluoro-phenyl) pyridinecarboxamide* (**12b**). A white solid; Yield: 62.3%; m.p.: 281.5–283.8 °C; ESI-MS [M + H] *m*/*z*: 560.1; ^1^H-NMR (DMSO-*d*_6_) δ 10.90 (s, 1H), 8.84 (d, *J* = 5.1 Hz, 1H), 8.45 (d, *J* = 1.2 Hz, 1H), 8.36 (d, *J* = 8.8 Hz, 2H), 8.09 (d, *J* = 8.9 Hz, 2H), 8.07 (dd, *J* = 5.1, 1.9 Hz, 1H), 7.84–7.76 (m, 2H), 7.66–7.60 (m, 1H), 7.42– 7.35 (m, 1H), 4.37 (s, 2H), 3.77 (d, *J* = 4.5 Hz, 4H), 3.58 (t, *J* = 6.4 Hz, 2H), 3.40 (t, *J* = 5.1 Hz, 6H).

*N-(4-(4-Morpholino-7,8-dihydro-6,6-dioxide-5H-thiopyrano[4,3-d]pyrimidin-2-yl)phenyl)-4-(2,4-difluorophenyl) picolinamide* (**12c**). A pale yellow solid; Yield: 63.0%; m.p.: 269.0–272.6 °C; ESI-MS [M + H] *m*/*z*: 578.1; ^1^H-NMR (DMSO-*d*_6_) δ 10.91 (s, 1H), 8.86 (d, *J* = 5.0 Hz, 1H), 8.36 (d, *J* = 8.7 Hz, 2H), 8.32 (s, 1H), 8.09 (d, *J* = 8.8 Hz, 2H), 7.90 (d, *J* = 5.1 Hz, 1H), 7.87–7.81 (m, 1H), 7.55–7.47 (m, 1H), 7.32 (t, *J* = 8.3 Hz, 1H), 4.37 (s, 2H), 3.78 (s, 4H), 3.57 (d, *J* = 6.6 Hz, 2H), 3.40 (t, *J* = 5.2 Hz, 6H).

*N-(4-(4-Morpholino-7,8-dihydro-6,6-dioxide-5H-thiopyrano[4,3-d]pyrimidin-2-yl)phenyl)-4-(4-trifluoro-methylphenyl) pyridinecarboxamide* (**12d**). A white solid; Yield: 62.4%; m.p.: >300.0 °C; ESI-MS [M + H] *m*/*z*: 610.1; ^1^H-NMR (DMSO-*d*_6_) δ 10.92 (s, 1H), 8.88 (d, *J* = 5.1 Hz, 1H), 8.49 (d, *J* = 1.2 Hz, 1H), 8.35 (s, 2H), 8.15 (d, *J* = 8.1 Hz, 2H), 8.11 (s, 3H), 7.94 (d, *J* = 8.3 Hz, 2H), 4.37 (s, 2H), 3.81–3.73 (m, 4H), 3.58 (t, *J* = 6.6 Hz, 2H), 3.40 (d, *J* = 4.5 Hz, 6H).

*N-(4-(4-Morpholino-7,8-dihydro-6,6-dioxide-5H-thiopyrano[4,3-d]pyrimidin-2-yl)phenyl)-4-(4-chloro-phenyl) pyridinecarboxamide* (**12e**). A pale yellow solid; Yield: 62.1%; m.p.: 266.8–268.8 °C; ESI-MS [M + H] *m*/*z*: 577.1; ^1^H-NMR (DMSO-*d*_6_) δ 10.90 (s, 1H), 8.83 (d, *J* = 5.1 Hz, 1H), 8.43 (d, *J* = 1.3 Hz, 1H), 8.36 (d, *J* = 8.8 Hz, 2H), 8.09 (d, *J* = 8.9 Hz, 2H), 8.04 (dd, *J* = 5.2, 1.9 Hz, 1H), 7.98–7.94 (m, 2H), 7.66–7.62 (m, 2H), 4.37 (s, 2H), 3.80–3.76 (m, 4H), 3.58 (t, *J* = 6.5 Hz, 2H), 3.40 (s, 6H).

*N-(4-(4-Morpholino-7,8-dihydro-6,6-dioxide-5H-thiopyrano[4,3-d]pyrimidin-2-yl)phenyl)-4-(4-fluoro-phenyl) pyridincarboxamide* (**12f**). A white solid; Yield: 63.3%; m.p.: >300.0 °C; ESI-MS [M + H] *m*/*z*: 560.2; ^1^H-NMR (DMSO-*d*_6_) δ 10.89 (s, 1H), 8.82 (d, *J* = 5.1 Hz, 1H), 8.42 (d, *J* = 1.4 Hz, 1H), 8.36 (d, *J* = 8.8 Hz, 2H), 8.09 (d, *J* = 8.9 Hz, 2H), 8.04–7.96 (m, 3H), 7.42 (t, *J* = 8.8 Hz, 2H), 4.37 (s, 2H), 3.77 (d, *J* = 4.8 Hz, 4H), 3.57 (t, *J* = 6.5 Hz, 2H), 3.40 (t, *J* = 5.2 Hz, 6H).

*N-(4-(4-Morpholino-7,8-dihydro-6,6-dioxide-5H-thiopyrano[4,3-d]pyrimidin-2-yl)phenyl)-4-(4-methylphenyl) pyridinecarboxamide* (**12g**). A pale yellow solid; Yield: 64.1%; m.p.: 290.2–293.1 °C; ESI-MS [M + H] *m*/*z*: 556.2; ^1^H-NMR (CDCl_3_) δ 10.88 (s, 1H), 8.78 (d, *J* = 5.1 Hz, 1H), 8.40 (s, 1H), 8.35 (d, *J* = 8.7 Hz, 2H), 8.08 (d, *J* = 8.7 Hz, 2H), 7.99 (d, *J* = 5.2 Hz, 1H), 7.81 (d, *J* = 8.0 Hz, 2H), 7.38 (d, *J* = 8.0 Hz, 2H), 4.36 (s, 2H), 3.76 (s, 4H), 3.57 (t, *J* = 6.4 Hz, 2H), 3.41–3.36 (m, 6H), 2.38 (s, 3H).

*N-(4-(4-Morpholino-7,8-dihydro-6,6-dioxide-5H-thiopyrano[4,3-d]pyrimidin-2-yl)phenyl)-4-(4-ethylphenyl) picolinamide* (**12h**). A pale yellow solid; Yield: 61.8%; m.p.: 276.8–279.1 °C; ESI-MS [M + H] *m*/*z*: 570.2; ^1^H-NMR (DMSO-*d*_6_) δ 10.88 (s, 1H), 8.79 (d, *J* = 5.1 Hz, 1H), 8.42 (d, *J* = 1.3 Hz, 1H), 8.36 (d, *J* = 8.8 Hz, 2H), 8.09 (d, *J* = 8.9 Hz, 2H), 8.01 (dd, *J* = 5.2, 1.9 Hz, 1H), 7.84 (d, *J* = 8.2 Hz, 2H), 7.42 (d, *J* = 8.2 Hz, 2H), 4.37 (s, 2H), 3.80–3.75 (m, 4H), 3.57 (t, *J* = 6.6 Hz, 2H), 3.40 (s, 6H), 2.70 (dd, *J* = 15.6, 8.1 Hz, 2H), 1.24 (t, 3H).

*N-(4-(4-Morpholino-7,8-dihydro-6,6-dioxide-5H-thiopyrano[4,3-d]pyrimidin-2-yl)phenyl)-4-(4-methoxy-phenyl) pyridinecarboxamide* (**12i**). A white solid; Yield: 63.1%; m.p.: 289.0–292.1 °C; ESI-MS [M + H] *m*/*z*: 572.2; ^1^H-NMR (DMSO-*d*_6_) δ 10.87 (s, 1H), 8.76 (d, *J* = 5.1 Hz, 1H), 8.40 (d, *J* = 1.3 Hz, 1H), 8.36 (d, *J* = 8.8 Hz, 2H), 8.09 (d, *J* = 8.8 Hz, 2H), 7.98 (dd, *J* = 5.2, 1.9 Hz, 1H), 7.90 (d, *J* = 8.9 Hz, 2H), 7.13 (d, *J* = 8.9 Hz, 2H), 4.37 (s, 2H), 3.85 (s, 3H), 3.80–3.76 (m, 4H), 3.57 (t, *J* = 6.5 Hz, 2H), 3.40 (t, *J* = 5.4 Hz, 6H).

*N-(4-(4-Morpholino-7,8-dihydro-6,6-dioxide-5H-thiopyrano[4,3-d]pyrimidin-2-yl)phenyl)-4-(3-methylphenyl) pyridinecarboxamide* (**12j**). A white solid; Yield: 62.5%; m.p.: 287.2–290.0 °C; ESI-MS [M + H] *m*/*z*: 556.1; ^1^H-NMR (CDCl_3_) δ 10.90 (s, 1H), 8.80 (d, *J* = 5.1 Hz, 1H), 8.41 (s, 1H), 8.35 (d, *J* = 8.6 Hz, 2H), 8.08 (d, *J* = 8.8 Hz, 2H), 8.00 (d, *J* = 5.2 Hz, 1H), 7.73 (s, 1H), 7.69 (d, *J* = 7.9 Hz, 1H), 7.46 (t, *J* = 7.8 Hz, 1H), 7.34 (d, *J* = 8.2 Hz, 1H), 4.36 (s, 2H), 3.77 (s, 4H), 3.57 (t, *J* = 6.6 Hz, 2H), 3.38 (d, *J* = 5.3 Hz, 6H), 2.42 (s, 3H).

*N-(4-(4-Morpholino-7,8-dihydro-5H-thiopyrano[4,3-d]pyrimidin-2-yl)phenyl)-6-(p-tolyl)pyrimidine-4-formamide* (**13a**). A pale yellow solid; Yield: 61.8%; m.p.: 257.0–259.7 °C; ESI-MS [M + H] *m*/*z*: 525.2; ^1^H-NMR (CDCl_3_) δ 10.23 (s, 1H), 9.34 (s, 1H), 8.77 (d, *J* = 8.4 Hz, 2H), 8.63 (s, 1H), 8.16 (d, *J* = 7.9 Hz, 2H), 8.03 (d, *J* = 8.4 Hz, 2H), 7.39 (d, *J* = 7.8 Hz, 2H), 3.97 (s, 4H), 3.90 (d, *J* = 13.0 Hz, 6H), 3.64 (s, 2H), 3.05 (s, 2H), 2.48 (s, 3H).

*N-(4-(4-Morpholino-7,8-dihydro-5H-thiopyrano[4,3-d]pyrimidin-2-yl)phenyl)-6-(p-methoxyphenyl) pyridine-4-carboxamide* (**13b**). A pale yellow solid; Yield: 64.1%; m.p.: 210.0–212.7 °C; ESI-MS [M + H] *m*/*z*: 541.2; ^1^H-NMR (DMSO-*d*_6_) δ 11.01 (s, 1H), 9.39 (s, 1H), 8.56 (s, 1H), 8.35 (t, *J* = 10.1 Hz, 4H), 8.09 (s, 2H), 7.16 (d, *J* = 8.6 Hz, 2H), 3.88 (s, 4H), 3.78 (s, 3H), 3.73 (s, 2H), 3.41 (s, 4H), 3.09 (s, 2H), 3.02 (s, 2H).

*N-(4-(4-Morpholino-7,8-dihydro-5H-thiopyrano[4,3-d]pyrimidin-2-yl)phenyl)-6-(4-trifluoromethylphenyl) pyridine-4-carboxamide* (**13c**). A pale yellow solid; Yield: 61.0%; m.p.: 244.2–245.8 °C; ESI-MS [M + H] *m*/*z*: 579.1; ^1^H-NMR (CDCl_3_) δ 10.11 (s, 1H), 9.40 (s, 1H), 8.69 (s, 1H), 8.59 (s, 2H), 8.36 (d, *J* = 8.1 Hz, 2H), 7.95 (d, *J* = 8.3 Hz, 2H), 7.83 (d, *J* = 8.4 Hz, 2H), 3.89 (s, 4H), 3.66 (s, 2H), 3.63 (s, 4H), 3.45 (s, 2H), 3.04 (s, 2H).

*N-(4-(4-Morpholino-7,8-dihydro-5H-thiopyrano[4,3-d]pyrimidin-2-yl)phenyl)-6-(4-fluorophenyl) pyridine-4-carboxamide* (**13d**). A pale yellow solid; Yield: 65.1%; m.p.: 237.0–239.3 °C; ESI-MS [M + H] *m*/*z*: 529.1; ^1^H-NMR (CDCl_3_) δ 10.16 (s, 1H), 9.33 (s, 1H), 8.70 (d, *J* = 8.6 Hz, 2H), 8.59 (s, 1H), 8.31–8.22 (m, 2H), 7.99 (d, *J* = 8.6 Hz, 2H), 7.24 (d, *J* = 8.6 Hz, 2H), 3.88 (s, 8H), 3.75 (s, 2H), 3.63 (s, 2H), 3.03 (s, 2H).

*N-(4-(4-Morpholino-7,8-dihydro-5H-thiopyrano[4,3-d]pyrimidin-2-yl)phenyl)-6-(4-bromophenyl) pyridine-4-carboxamide* (**13e**). A yellow solid; Yield: 61.4%; m.p.: 251.8–255.0 °C; ESI-MS [M + H] *m*/*z*: 590.1; ^1^H-NMR (CDCl_3_) δ 10.19 (s, 1H), 9.35 (s, 1H), 8.75 (s, 2H), 8.61 (s, 1H), 8.12 (d, *J* = 8.4 Hz, 2H), 8.01 (d, *J* = 8.2 Hz, 2H), 7.70 (d, *J* = 8.3 Hz, 2H), 3.96 (s, 4H), 3.90 (s, 4H), 3.87–3.81 (m, 2H), 3.63 (s, 2H), 3.04 (s, 2H).

*N-(4-(4-Morpholino-7,8-dihydro-5H-thiopyrano[4,3-d]pyrimidin-2-yl)phenyl)-6-(4-chlorophenyl) pyridine-4-carboxamide* (**13f**). A yellow solid; Yield: 61.5%; m.p.: 281.8–285.0 °C; ESI-MS [M + H] *m*/*z*: 546.1; ^1^H-NMR (DMSO-*d*_6_) δ 11.05 (s, 1H), 9.49 (s, 1H), 8.65 (s, 1H), 8.37 (s, 4H), 8.07 (d, *J* = 8.4 Hz, 2H), 7.68 (d, *J* = 8.6 Hz, 2H), 3.77 (s, 4H), 3.72 (s, 2H), 3.40 (s, 4H), 3.09 (s, 2H), 3.01 (s, 2H).

*N-(4-(4-Morpholino-7,8-dihydro-5H-thiopyrano[4,3-d]pyrimidin-2-yl)phenyl)-6-phenylpyridine-4-carboxamide* (**13g**). A yellow solid; Yield: 63.8%; m.p.: 250.1–252.3 °C; ESI-MS [M + H] *m*/*z*: 511.2; ^1^H-NMR (DMSO-*d*_6_) δ 11.04 (s, 1H), 9.48 (s, 1H), 8.63 (s, 1H), 8.35 (t, *J* = 7.3 Hz, 4H), 8.07 (d, *J* = 8.7 Hz, 2H), 7.62 (d, *J* = 6.6 Hz, 3H), 3.77 (s, 4H), 3.72 (s, 2H), 3.39 (s, 4H), 3.08 (d, *J* = 5.6 Hz, 2H), 3.02 (d, *J* = 5.6 Hz, 2H).

*N-(4-(4-Morpholino-7,8-dihydro-6,6-dioxide-5H-thiopyrano[4,3-d]pyrimidin-2-yl)phenyl)-6-(p-tolyl) pyrimidine-4-carboxamide* (**14a**). A pale yellow solid; Yield: 62.7%; m.p.: 260.1–263.0 °C; ESI-MS [M + H] *m*/*z*: 557.2; ^1^H-NMR (DMSO-*d*_6_) δ 11.05 (s, 1H), 9.43 (s, 1H), 8.58 (s, 1H), 8.37 (d, *J* = 8.6 Hz, 2H), 8.24 (d, *J* = 8.0 Hz, 2H), 8.09 (d, *J* = 8.6 Hz, 2H), 7.41 (d, *J* = 7.9 Hz, 2H), 4.38 (s, 2H), 3.78 (s, 4H), 3.59 (t, *J* = 6.0 Hz, 2H), 3.40 (d, *J* = 14.5 Hz, 6H), 2.41 (s, 3H).

*N-(4-(4-Morpholino-7,8-dihydro-6,6-dioxide-5H-thiopyrano[4,3-d]pyrimidin-2-yl)phenyl)-6-(p-methoxyphenyl) pyridine-4-carboxamide* (**14b**). A yellow solid; Yield: 61.7%; m.p.: 277.0–279.5 °C; ESI-MS [M + H] *m*/*z*: 573.1; ^1^H-NMR (DMSO-*d*_6_) δ 11.03 (s, 1H), 9.37 (s, 1H), 8.53 (s, 1H), 8.35 (d, *J* = 8.4 Hz, 2H), 8.30 (d, *J* = 9.0 Hz, 2H), 8.07 (d, *J* = 8.7 Hz, 2H), 7.12 (d, *J* = 8.9 Hz, 2H), 4.35 (s, 2H), 3.85 (s, 3H), 3.84–3.80 (m, 2H), 3.75 (s, 4H), 3.56 (s, 6H).

*N-(4-(4-Morpholino-7,8-dihydro-6,6-dioxide-5H-thiopyrano[4,3-d]pyrimidin-2-yl)phenyl)-6-(4-trifluoro-methylphenyl) pyridine-4-carboxamide* (**14c**). A pale yellow solid; Yield: 62.5%; m.p.: 287.1–289.2 °C; ESI-MS [M + H] *m*/*z*: 611.1; ^1^H-NMR (DMSO-*d*_6_) δ 11.12 (s, 1H), 9.53 (s, 1H), 8.71 (s, 1H), 8.53 (d, *J* = 7.9 Hz, 2H), 8.35 (d, *J* = 8.6 Hz, 2H), 8.08 (d, *J* = 8.6 Hz, 2H), 7.95 (d, *J* = 7.3 Hz, 2H), 4.36 (s, 2H), 3.75 (s, 6H), 3.54 (s, 6H).

*N-(4-(4-Morpholino-7,8-dihydro-6,6-dioxide-5H-thiopyrano[4,3-d]pyrimidin-2-yl)phenyl)-6-(4-fluoro-phenyl) pyridine-4-carboxamide* (**14d**). A yellow solid; Yield: 61.3%; m.p.: >300 °C; ESI-MS [M + H] *m*/*z*: 561.1; ^1^H-NMR (DMSO-*d*_6_) δ 11.07 (s, 1H), 9.44 (s, 1H), 8.61 (s, 1H), 8.40 (s, 2H), 8.35 (d, *J* = 8.6 Hz, 2H), 8.07 (d, *J* = 8.2 Hz, 2H), 7.42 (s, 2H), 4.35 (s, 2H), 3.75 (s, 6H), 3.56 (s, 6H).

*N-(4-(4-Morpholino-7,8-dihydro-6,6-dioxide-5H-thiopyrano[4,3-d]pyrimidin-2-yl)phenyl)-6-(4-bromophenyl) pyridine-4-carboxamide* (**14e**). A yellow solid; Yield: 67.1%; m.p.: >300 °C; ESI-MS [M + H] *m/z*: 622.1; ^1^H-NMR (DMSO-*d*_6_) δ 11.06 (s, 1H), 9.46 (s, 1H), 8.63 (s, 1H), 8.34 (d, *J* = 8.9 Hz, 2H), 8.27 (d, *J* = 8.6 Hz, 2H), 8.06 (d, *J* = 8.8 Hz, 2H), 7.79 (d, *J* = 8.6 Hz, 2H), 4.35 (s, 2H), 3.75 (s, 4H), 3.55 (d, *J* = 6.2 Hz, 2H), 3.38 (s, 6H).

*N-(4-(4-Morpholino-7,8-dihydro-6,6-dioxide-5H-thiopyrano[4,3-d]pyrimidin-2-yl)phenyl)-6-(4-chloro-phenyl) pyridine-4-carboxamide* (**14f**). A white solid; Yield: 61.4%; m.p.: >300 °C; ESI-MS [M + H] *m*/*z*: 578.1; ^1^H-NMR (DMSO-*d*_6_) δ 11.08 (s, 1H), 9.46 (s, 1H), 8.63 (s, 1H), 8.35 (d, *J* = 8.5 Hz, 4H), 8.07 (d, *J* = 8.7 Hz, 2H), 7.65 (d, *J* = 8.6 Hz, 2H), 4.35 (s, 2H), 3.75 (s, 4H), 3.55 (d, *J* = 6.4 Hz, 2H), 3.38 (s, 6H).

*N-(4-(4-Morpholino-7,8-dihydro-6,6-dioxide-5H-thiopyrano[4,3-d]pyrimidin-2-yl)phenyl)-6-phenylpyridine-4-carboxamide* (**14g**). A green solid; Yield: 62.8%; m.p.: 294.2–296.6 °C; ESI-MS [M + H] *m*/*z*: 543.2; ^1^H-NMR (CDCl_3_) δ 10.12 (s, 1H), 9.34 (s, 1H), 8.67 (s, 1H), 8.49 (d, *J* = 7.8 Hz, 2H), 8.25 (s, 2H), 7.93 (d, *J* = 8.0 Hz, 2H), 7.59 (s, 2H), 7.58–7.55 (m, 1H), 4.18 (s, 2H), 3.89 (s, 4H), 3.61 (s, 2H), 3.45 (s, 6H).

^1^H-NMR spectra for representative final compounds (**11a**, **11c**, **12d**, **12j**, **13b**, **13d**, **14b** and **14d**) can be seen in Figures S1–S8 in Supplementary Materials.

### 3.3. PI3Kα Kinase Assay

The selected compound **12h** was tested for its activity against PI3Ka using a Kinase-Glo^®^Luminescent Kinase Assay (Promega, Madison, WI, USA), with GDC-0941 and PI103 as positive control. The kinase reaction is done in a 384-well black plate. Each well is loaded with 50 µL of test items (in 90% DMSO) and 5 µL reaction buffer containing 10 µg/mL PI substrate (L-α-phosphatidylinositol; Avanti Polar Lipids (Avanti Polar Lipids, Inc., Alabaster, AL, USA); prepared in 3% octyl-glucoside) and the PI3Ka protein 10 nM is then added to it. The reaction is started by the addition of 5 µL of 1 µM ATP prepared in the reaction buffer and is incubated for 60 min for p110a. It is terminated by the addition of 10 µL Kinase-Glo buffer. The plates are then read in a Synergy 2 reader (BioTek, Winooski, VT, USA) for luminescence detection. The assay was repeated two times [3,7].

### 3.4. Cytotoxicity Assay In Vitro

The in vitro cytotoxic activities of all the compounds **11a**–**11j**, **12a**–**12j**, **13a**–**13g** and **14a**–**14g** was evaluated with A549, PC-3 and MCF-7 cell lines by the standard MTT assay, with GDC-0941 as positive control. The cancer cell lines were cultured in minimum essential medium (MEM) supplement with 10% fetal bovine serum (FBS). Approximately 4 × 10^3^ cells, suspended in MEM medium, were plated onto each well of a 96-well plate and incubated in 5% CO_2_ at 37 °C for 24 h. The test compounds at indicated final concentrations were added to the culture medium and the cell cultures were continued for 72 h. Fresh MTT was added to each well at a terminal concentration of 5 µg/mL and incubated with cells at 37 °C for 4 h. The formazan crystals were dissolved in 100 µL DMSO each well, and the absorbency at 492 nm (for absorbance of MTT formazan) and 630 nm (for the reference wavelength) was measured with an ELISA reader. All of the compounds were tested three times in each of the cell lines. The results expressed as inhibition rates or IC_50_ (half-maximal inhibitory concentration) were the averages of two determinations and calculated by using the Bacus Laboratories Inc. Slide Scanner (Bliss) software (the Bacus Laboratories Inc. Slide Scanner (BLISS) system, Lombard, IL, USA) [3,7].

### 3.5. Docking Studies

For docking purposes, we prepared the receptor proteins PDB ID code: 4L23 (PI3Ka). The threedimensional structure of the PI3Ka were obtained from RCSB Protein Data Bank. We built a small organic molecule set (compound **12h**) and used the Gasteiger–Huckel method to optimize the molecular force field and structure. Hydrogen atoms were added to the structure allowing for appropriate ionization at physiological pH. First of all, extract ligand substructure, then remove water and excess structure, finally, add hydrogens and fix sidechain amides. The protonated state of several important residues, such as GLU849, ASP810, ASP933, VAL851 and LYS802, were adjusted by using SYBYL6.9.1 (Tripos, St. Louis, MO, USA) in favor of forming reasonable hydrogen bond with the ligand. Molecular docking analysis was carried out by the SURFLEX-DOCK module of SYBYL 6.9.1 package to explore the binding model for the active site of PI3Ka with its ligand. All atoms located within the range of 5.0 Å from any atom of the cofactor were selected into the active site, and the corresponding amino acid residue was, therefore, involved into the active site if only one of its atoms was selected. Other default parameters were adopted in the SURFLEX-DOCK calculations. All calculations were performed on a Silicon Graphics workstation. Lastly, docking results and the optimized molecular docking model with the receptor proteins was obtained [3,7,9].

## 4. Conclusions

In summary, four series of 4-morpholino-7,8-dihydro-5*H*-thiopyrano[4,3-*d*]pyrimidine derivatives bearing phenylpyridine/phenylpyrimidine carboxamide scaffolds were designed, synthesized and evaluated for antitumor activity against three cancer cell lines and PI3Kα kinase (only compound **12h**) in vitro. The pharmacological results indicated that eleven of the compounds showed moderate cytotoxicity activity against the three cancer cell lines. In particular, the activity of the most promising compound **12h** was equal to the positive control GDC-0941 against A549, with n IC_50_ value of 8.37 ± 0.10. Structure-activity relationships (SARs) indicated that the introduction of the phenylpyridine carboxamide scaffold was more favorable than the introduction of a phenylpyrimidine carboxamide scaffold for the activity. In general, the oxidation of a sulfur atom played no significant impact on the activity in the phenylpyridine carboxamide series, however, it could be appropriate to enhance the anti-tumor activity in the phenylpyrimidine carboxamide series. What’s more, various types of substituents impacted differently on the activity of the four series of compounds. Furthermore, the position of substituents on aryl group played a slight role in the activity of the first two series of compounds. Further studies will be carried out in the near future.

## Figures and Tables

**Table 1:**
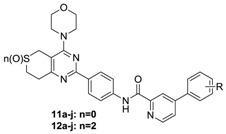
Structures and cytotoxicity of compounds **11a**–**j** and **12a**–**j**.

Compound	n	R	IC_50_ (μM) ^a^		
			A549	PC-3	MCF-7
**11a**	0	H	NA ^c^	NA	NA
**11b**	0	3-fluoro	48.08 ± 0.13	60.29 ± 1.24	51.67 ± 1.56
**11c**	0	2,4-difluoro	NA	NA	NA
**11d**	0	4-trifluoromethyl	43.46 ± 0.12	NA	50.28 ± 2.18
**11e**	0	4-chloro	19.28 ± 0.86	23.20 ± 0.76	20.88 ± 1.02
**11f**	0	4-fluoro	NA	NA	NA
**11g**	0	4-methyl	NA	NA	NA
**11h**	0	4-ethyl	NA	NA	NA
**11i**	0	4-methoxy	27.68 ± 0.10	65.66 ± 0.61	32.37 ± 1.84
**11j**	0	3-ethyl	11.59 ± 0.11	15.29 ± 0.83	12.43 ± 0.96
**12a**	2	H	NA	74.27 ± 0.08	NA
**12b**	2	3-fluoro	NA	NA	NA
**12c**	2	2,4-difluoro	NA	NA	NA
**12d**	2	4-trifluoromethyl	NA	NA	NA
**12e**	2	4-chloro	NA	NA	NA
**12f**	2	4-fluoro	NA	NA	NA
**12g**	2	4-methyl	14.51 ± 0.10	64.71 ± 0.17	28.92 ± 1.29
**12h**	2	4-ethyl	8.37 ± 0.10	11.34 ± 0.11	9.26 ± 0.82
**12i**	2	4-methoxy	NA	NA	NA
**12j**	2	3-ethyl	NA	NA	NA
**GDC-0941 ^b^**	-	-	6.99 ± 0.21	0.20 ± 0.08	0.07 ± 0.03

^a^ The values are an average of two separate determinations; ^b^ Used as a positive controls; ^c^ No Activity.

**Table 2:**
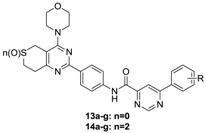
Structures and cytotoxicity of compounds **13a**–**g** and **14a**–**g**.

Compound	n	R	IC_50_ (μM) ^a^		
			A549	PC-3	MCF-7
**13a**	0	4-ethyl	NA ^c^	NA	NA
**13b**	0	4-methoxy	NA	NA	NA
**13c**	0	4-trifluoromethyl	NA	NA	NA
**13d**	0	4-fluoro	NA	NA	NA
**13e**	0	4-bromo	NA	NA	NA
**13f**	0	4-chloro	NA	NA	NA
**13g**	0	H	NA	NA	NA
**14a**	2	4-ethyl	NA	81.69 ± 4.92	38.71 ± 1.72
**14b**	2	4-methoxy	NA	NA	89.64 ± 0.91
**14c**	2	4-trifluoromethyl	NA	NA	NA
**14d**	2	4-fluoro	NA	NA	NA
**14e**	2	4-bromo	NA	NA	27.37 ± 1.27
**14f**	2	4-chloro	NA	NA	NA
**14g**	2	H	NA	NA	NA
**GDC-0941 ^b^**	-	-	6.99 ± 0.21	0.20 ± 0.08	0.07 ± 0.03

^a^ The values are an average of two separate determinations; ^b^ Used as a positive controls; ^c^ No Activity.

**Table 3:** PI3Ka kinase activity of selected compounds 12 h and positive controls.

Compound	IC_50_^a^ (µM)
	PI3Kα
**12h**	7.386 ± 0.194
**GDC-0941** ^b^	0.003
**PI-103** ^b^	0.075 ± 0.018

^a^ The values are an average of two separate determinations; ^b^ Used as a positive controls.

## Supplementary Materials

Supplementary materials are available online at http://www.mdpi.com/1420-3049/21/11/1447/s1.
