# Supplementary material for: Synthesis and Biological Evaluation of Novel 4-Morpholino-7,8-dihydro-5H-thiopyrano[4,3-d]pyrimidine Derivatives Bearing Phenylpyridine/ Phenylpyrimidine-Carboxamides
Source: Molecules. 2016 Oct 31;21(11):1447. doi: 10.3390/molecules21111447 (PMC6273168; doi:10.3390/molecules21111447)
Supplement: Supplementary file 1 [file molecules-21-01447-s001.pdf]

Supplementary Materials: Synthesis and Biological Evaluation of Novel 4-Morpholino-7,8-dihydro-5*H*-thiopyrano[4,3-*d*]pyrimidine Derivatives Bearing Phenylpyridine-/Phenylpyrimidine-Carboxamide

Huimin Liu, Wenhui Wang, Chengyu Sun, Caolin Wang and Wufu Zhu and Pengwu Zheng

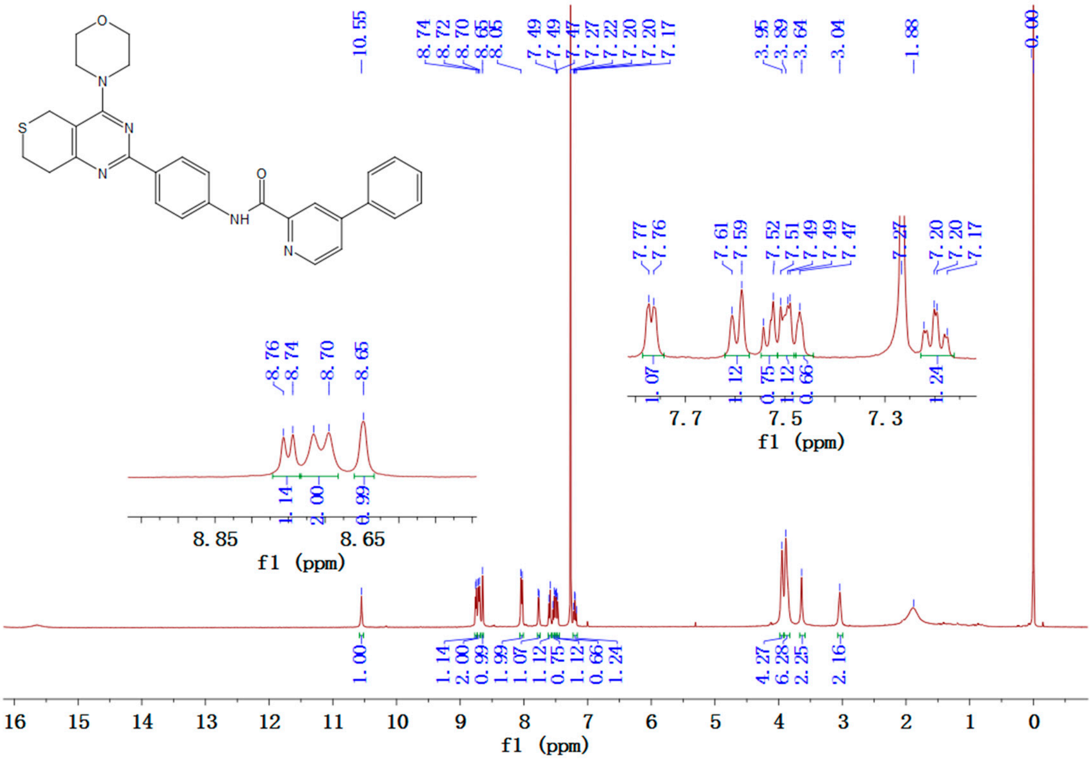

Figure 1. ^{1}H NMR spectrum of compound **11a**.

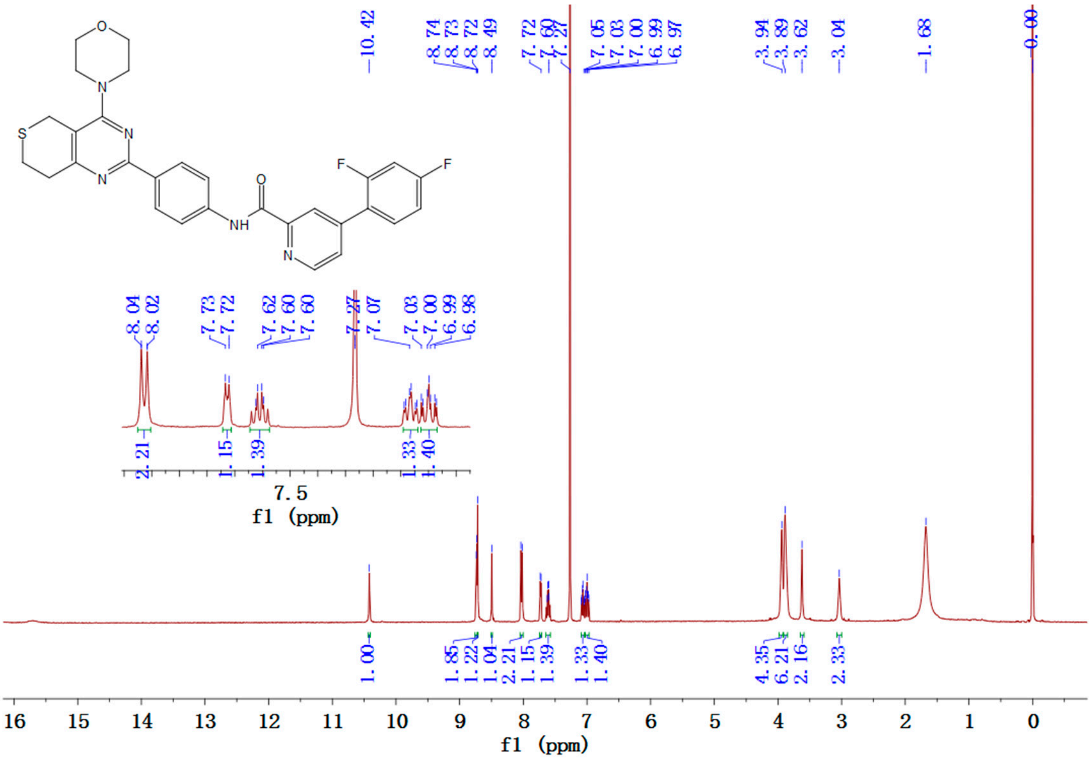

Figure 2. ^{1}H NMR spectrum of compound **11c**.

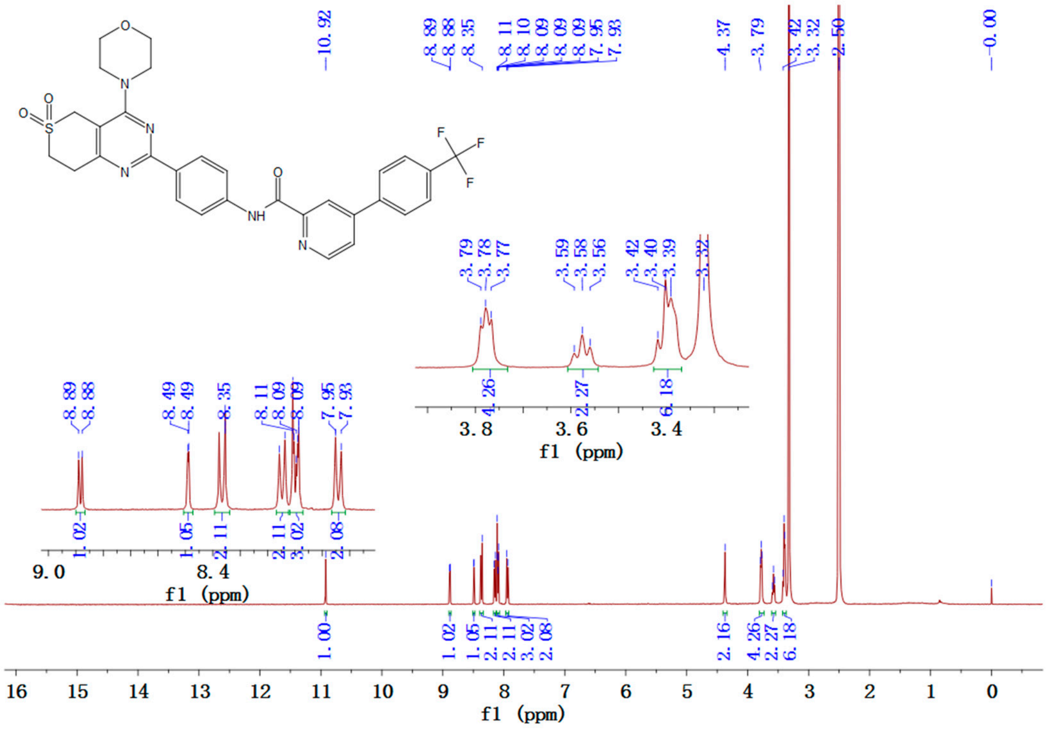

Figure 3. ^{1}H NMR spectrum of compound **12d**.

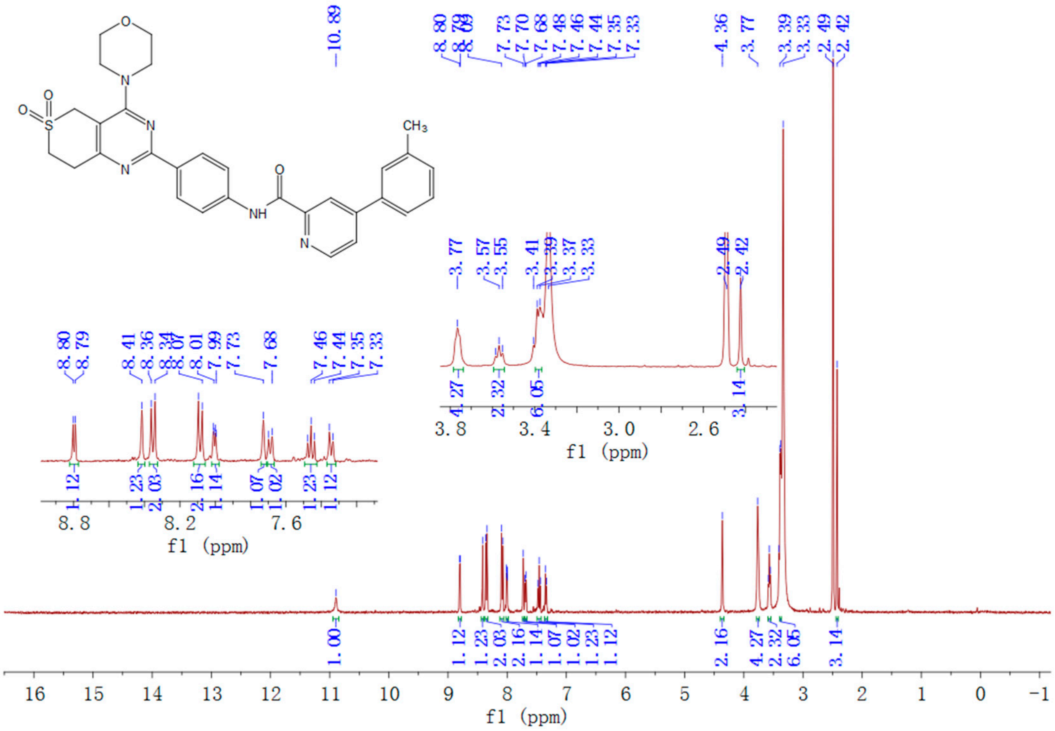

Figure 4. ^{1}H NMR spectrum of compound **12j**.

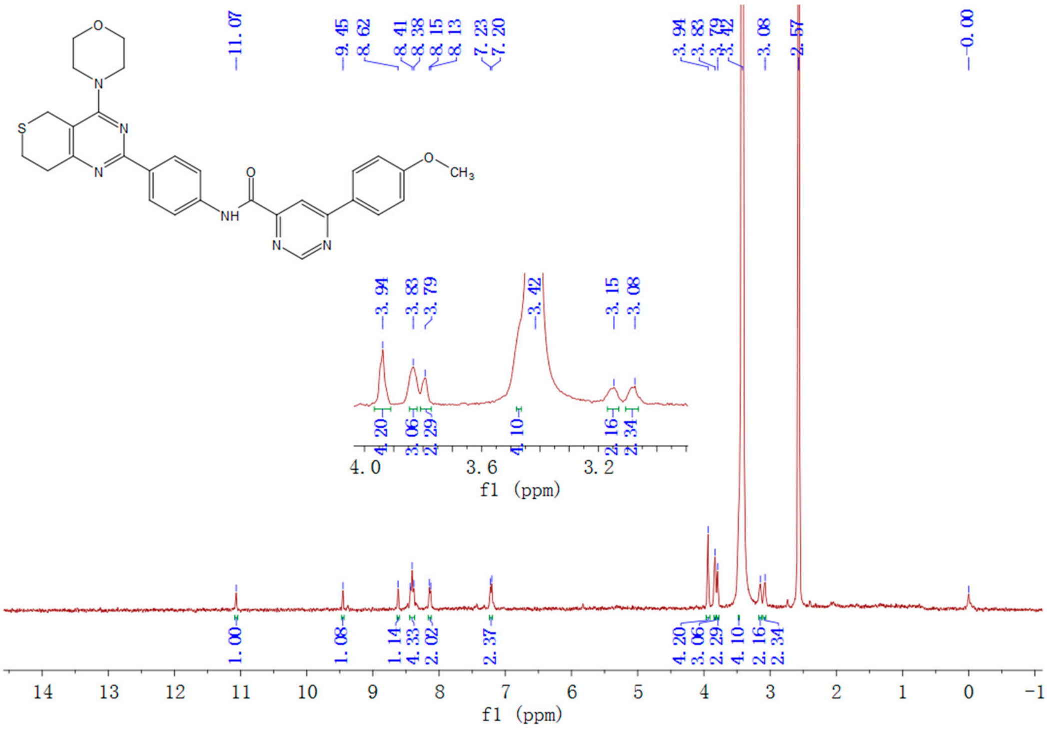

Figure 5. ^{1}H NMR spectrum of compound **13b**.

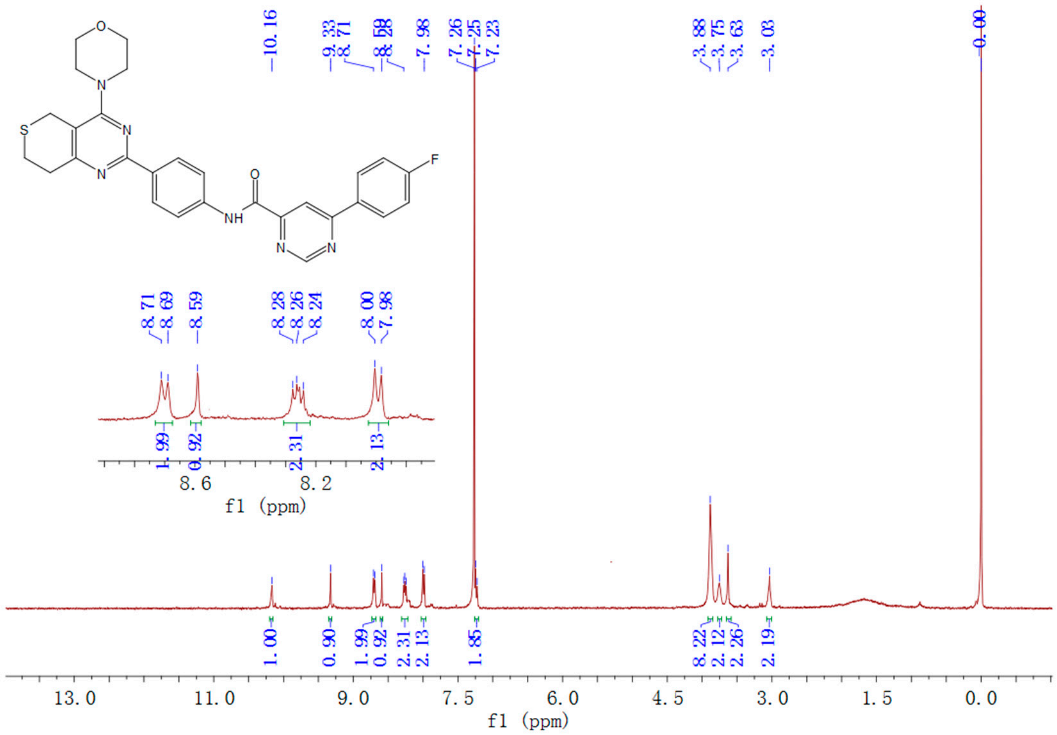

Figure 6. ^{1}H NMR spectrum of compound **13d**.

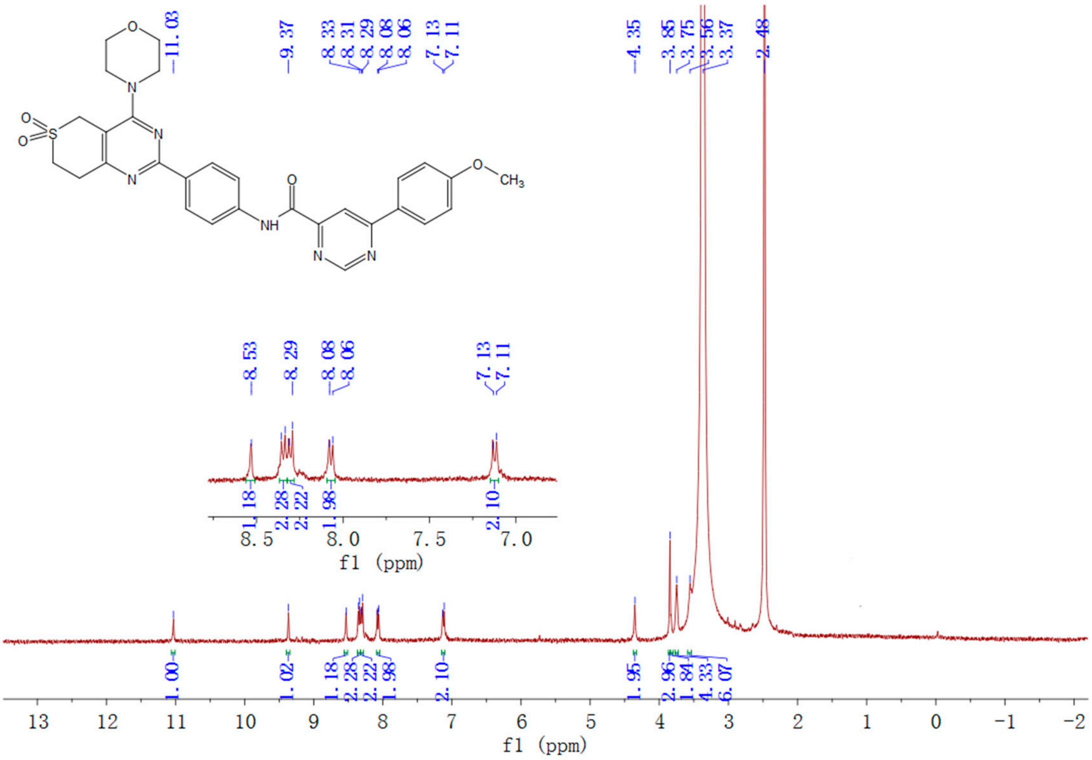

Figure 7. ^{1}H NMR spectrum of compound **14b**.

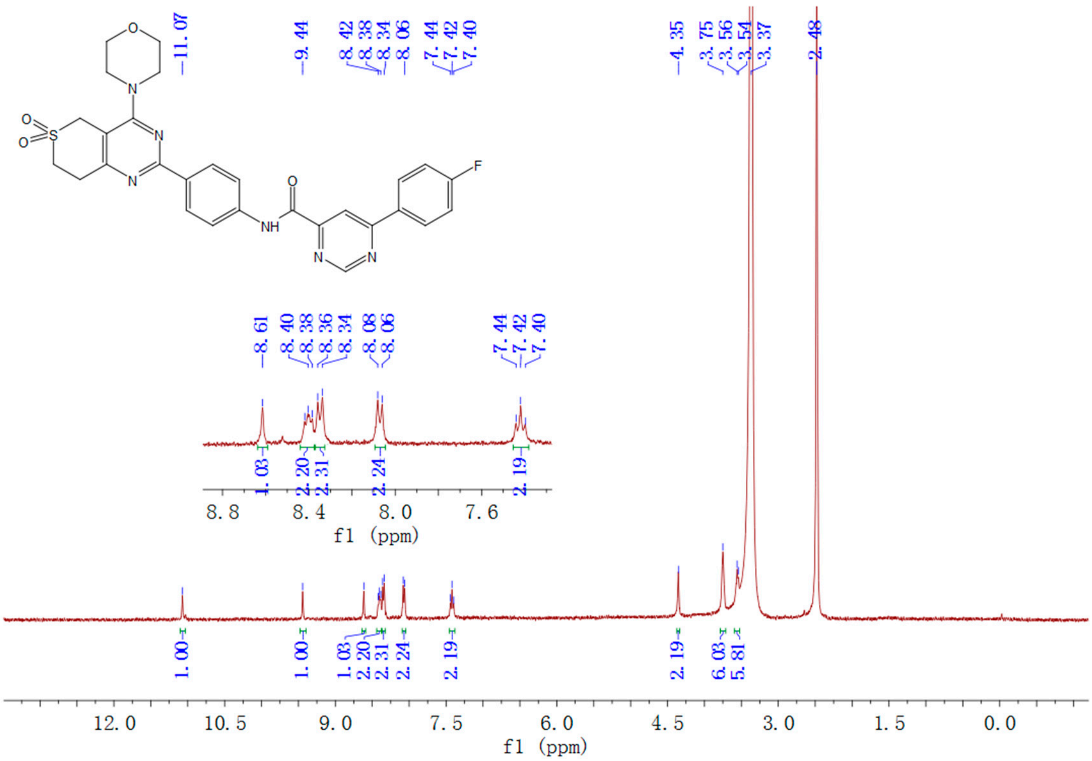

Figure 8. ^{1}H NMR spectrum of compound **14d**.
